# What is the Optimal Way to Assess *Meloidogyne* Spp. Reproduction in Greenhouse Pot Experiments?

**DOI:** 10.2478/jofnem-2022-0012

**Published:** 2022-05-18

**Authors:** Olivia Filialuna, Catherine Wram, Inga Zasada

**Affiliations:** 1 USDA-ARS, Horticultural Crops Research Unit, Corvallis, OR, 97330, Telephone 541-738-4051, Fax 541-738-4025

**Keywords:** greenhouse, inoculation density, life stage, reproduction factor, root-knot nematode, technique

## Abstract

Often research efforts that address both the practical concerns of managing *Meloidogyne* spp. and understanding their basic biology involve greenhouse reproduction assays. However, there is little consensus in regards to what parameters should be used to conduct greenhouse assays. The goal of this research was to evaluate how pot size, *Meloidogyne* spp. inoculation life stage, inoculation density, and time of assay impacted final reproduction factor (RF = initial nematode density/final nematode density) values. In experiments with *M. incognita*, the factor of the pot size mattered, with higher RF values in pots containing 500 g soil *vs.* pots with 100 g soil; larger pots containing 3,000 g soil did not have RF values different from the aforementioned sizes. Inoculating with *M. incognita* J2 resulted in RF values on average of >2 fold higher then when inoculating with eggs at comparable densities. Inoculation density of *M. incognita* did not have an impact on final *M. incognita* RF values. In experiments that considered time of assay, three species were evaluated: *M. incognita, M. chitwoodi*, and *M. hapla.* There was no difference in *M. incognita* RF values when assays were conducted for 5 wk, 6 wk, 7 wk, and 8 wk. However, a longer assay time resulted in higher RF values for *M. hapla* and *M. chitwoodi*, with at least a 7 week assay required. In conclusion, a moderate pot size (500 g of soil) inoculated with *M. incognita* J2 resulted in maximum RF values. The length of the assay required will depend on the *Meloidogyne* spp. in question, with longer duration assays required for *M. hapla* and *M. chitwoodi* than for *M. incognita.*

Of the >4,000 plant-parasitic nematode species, those in the genus *Meloidogyne* are some of the most important (Perry *et al*., 2009; Jones *et al*., 2013). In particular, *M. incognita, M. hapla*, and *M. chitwoodi* are of significant concern for growers of a wide variety of crops, including annual and perennial plants, due to their devastating effects on yield and crop quality (Perry *et al*., 2009). *M. incognita* is widespread throughout the Americas and Africa, and present in much of Europe and Asia, while *M. hapla* is found in most temperate regions throughout the world (CABI, 2021a, 2021b). *M. chitwoodi* is endemic in the western USA, Argentina, and Chile, where it is of major concern for the production of potato (CABI, 2021c).

*Meloidogyne* spp. are often the subject of research for the development of plant-parasitic nematode management strategies, improvement of molecular nematode identification efforts, and understanding plant and plant-parasitic nematode interactions. Due to *Meloidogyne* spp. short generation time, the development of a unique host feeding site, ubiquitous nature, and host range that includes most vascular plants, they are an ideal model plant-parasitic nematode (Perry *et al*., 2009). Often research efforts that address both the practical concerns of managing *Meloidogyne* spp. and understanding their basic biology involve greenhouse reproduction assays. However, there is little consensus in regards to what parameters should be used to conduct greenhouse assays, including the optimal length of time an assay should run, what pot size is best suited for nematode reproduction, initial inoculation density, and what life stage should be used for inoculum.

In previous studies, researchers have utilized a range of different pot sizes, from a 473 mL cup to a 19 L cylindrical pot (Table 1). Duration of reproduction assays with *Meloidogyne* spp. has also varied from as little as 1 mon to >6 mon (Table 1). The use of *Meloidogyne* spp. eggs and J2 also varied, with inoculation densities from as little as 200 J2 to over 16,000 eggs per pot (Table 1). This range in pot size, inoculation density, and assay duration, has resulted in a wide range of reproduction factor (RF = final population density/initial population density) values, with experiments yielding RF values as small as 0.017 to >400. For example, an assay with an inoculation density of 1,275 *M. chitwoodi* J2 per plant in a 437 mL cup, and an experimental duration of 10 wk, yielded a RF value of 16 (Ferris *et al*., 1993). When 1,000 *M. chitwoodi* eggs were used to inoculate 2 L of soil and the experiment was conducted for 13 wk, a RF value of 442 was reported (Santo and O’Bannon, 1981). Some of this dramatic variation in RF values could be due to virulence of the *M. chitwoodi* population or susceptibility of the host; however, this variation could also be attributed to pot size, initial inoculation density, and assay duration. Understanding how these factors influence the reproductive capacity of *Meloidogyne* spp. is important for optimizing the greenhouse assay methodology, but is also crucial to consider when comparing results across research studies.

**Table 1:** Summary of experiments and the variables assessed.

	**Variables assessed**				
	**Life stage^a^**	**Takedown wk^b^**	**Pot size (g)**	**Inoculation density^c^**	**Species**
**Experiment 1**	Eggs, J2	8	100, 500, 3,000	0.5, 1, 2	*M. incognita*
**Experiment 2**	Eggs, J2	5, 6, 7, 8	500	1	*M. incognita*
**Experiment 3**	Eggs	5, 6, 7, 8	500	1	*M. chitwoodi, M. hapla, M. incognita*

Experiment 1 investigated the influence of life stage, pot size, and inoculation density on *Meloidogyne incognita* reproduction. Experiment 2 investigated the influence of length of assay based on time since inoculation on *M. incognit*a reproduction factor. Experiment 3 investigated the influence of assay length on reproduction of *M. chitwoodi*, *M. hapla*, and *M. incognita*.

a Life stage of inoculum used in each experiment.

b Week post inoculation when plants were destructively harvested.

c Inoculation density per gram soil.

Often when designing and implementing a greenhouse assay, the availability of inoculum can be a limiting factor as plant-parasitic nematodes can be difficult to maintain in culture or because there is a limited amount of material. Time constraints, accessibility, and cost of soil and pots to use for assays also play an important role in designing greenhouse experiments. In addition to the various logistics of setting up an experiment, it is important to take into account the differences in life cycle duration of different *Meloidogyne* spp. Considering these different factors and the variability of findings in the literature for *Meloidogyne* spp. pot experiments, the goal of this study was to determine the optimal and fastest methodology for conducting greenhouse assays with three *Meloidogyne* species. The objectives were to explore the impact of different parameters using *M. incognita* by (i) assessing the impact of inoculum life stage and density in three commonly used pot sizes and (ii) measuring the effects of inoculum life stage and assay duration on *M. incognita* reproduction. The third objective was to evaluate greenhouse assay duration on the reproduction of *M. hapla* and *M. chitwoodi*.

## Materials and Methods

### Nematode inoculum

Three *Meloidogyne* spp. were used in the experiments. *M. incognita* was collected from grape (*Vitis vinifera*) in Parlier, CA, *M. hapla* was collected from grape in Alderdale, WA, and *M. chitwoodi* was collected from a potato (*Solanum tuberosum*) in Prosser, WA. Single female lines were established for each population by adding nematode infested soil to a pot and planting tomato (*Solanum lycopersicum*) “Rutgers.” The identity of the populations was confirmed by molecular analysis by the North Carolina Department of Agriculture & Consumer Services (Raleigh, NC). After approximately 8 wk, the plants were destructively harvested and single egg masses were transferred to a new tomato plant. Cultures were then continuously maintained on tomato “Rutgers,” using 12–15 eggs masses to inoculate the fresh culture tomato plants. To extract the eggs for use in experiments, tomato plants were destructively harvested and the roots rinsed free of soil. Eggs were extracted by shaking the root system in a 0.3% NaOH solution for 3 min and then passing the solution over a 500 mesh sieve to collect eggs. To obtain *Meloidogyne* spp. J2 inoculum, extracted eggs were placed on a hatching chamber for 24–72 hr, and hatched J2 were collected every 24 hr. *Meloidogyne* spp. eggs and J2 were kept at 4°C until their use in experiments. *Meloidogyne* spp. egg and J2 densities were adjusted in water to achieve the desired inoculation densities needed for each experiment.

### Experiment 1: Impact of the volume of soil, initial inoculation density, and life stage used for inoculation on M. incognita reproduction

The experiment consisted of 72 experimental units (pots), and was a factorial experiment with the following factors: three volumes of soil (100 g, 500 g, and 3,000 g), three initial inoculation densities (0.5, 1.0, and 2.0 *M. incognita*/g soil), and two life stages (egg and J2) (Table 2). All factor combinations were replicated four times, and pots were arranged in a completely randomized design; the experiment was conducted twice. For the volume of soil factor, the pot size and type varied: for 100 g, soil was placed in a cell of a 0.12 L 6-pack seedling tray; for 500 g, soil was placed in a 10 cm^2^ plastic 0.6 L pot; and, for 3,000 g, soil was placed in a round 3.7 L plastic pot. For each pot, a 3- to 4-week-old tomato “Rutgers” was transplanted into a steam-pasteurized 1:1 sand:loam soil mix. At planting, the desired initial density/life stage was applied directly to the root system in 4 mL water. Immediately after inoculation, the plants were watered with enough 20-20-20 NPK starter fertilizer (J.R. Peters, Allentown, PA) to completely wet the soil but avoid loss of water from the bottom of the pots. Plants were maintained in a greenhouse under long-day conditions (16 hr photoperiod), with 26/18°C day/night temperatures. Plants were fertigated weekly with 20-8.8-16.6 NPK (J.R. Peters). The experiment was terminated 8 wk after planting and inoculation. The tops were removed and discarded. The contents of the pot were then emptied onto a tray and roots separated from soil, and the soil discarded. Collected roots were rinsed free of soil and eggs extracted by shaking the root system in 0.6% NaOH solution for 3 min and then passing the solution over a 500 mesh sieve to collect eggs. After extraction, roots were placed in a drying oven at 65°C for a week, and then weighed. Collected eggs were enumerated using a Leica DM IL inverted microscope (Leica Microsystems, Wetzlar, Germany).

**Table 2:** Summary of previous *Meloidogyne* species reproduction assay studies.

**Study**	**Species**	**Pot size^a^**	**Inoculation density^b^**	**Assay duration (degree day or time)**	**Experimental temperature**	**Reproduction factor or Gall rating^c^**
Pinkerton *et al*. (1991)	*M. chitwoodi*	19 L	150 eggs/250 cm^3^	1,750 DD	21°C to 22°C	20
Pinkerton *et al*. (1991)	*M. chitwoodi*	19 L	150 eggs/250 cm^3^	2,000 DD	21°C to 22°C	120
Ferris *et al*. (1993)	*M. chitwoodi*	473 mL	1,275 J2	10 wk	N/A	16.07
Ferris *et al*. (1993)	*M. chitwoodi*	473 mL	1,000 J2	90 d	N/A	2.6–16.5
Mojtahedi *et al*. (1988)	*M. chitwoodi* and *M. hapla*	10 cm d	5,000 eggs	55 d	22°C to 28°C	47.3
Santo and O’Bannon (1981)	*M. chitwoodi* and *M. hapla*	18 cm d	1,000 or 10,000 eggs	13 wk	15, 20, 25, and 30°C	*M. chitwoodi*: (6.9–442.8) *M. hapla*: (0.45–34)
Gaspard *et al*. (1990)	*M. incognita*	172 cm^3^	800 J2 to 1,500 J2	1 mon	500 DD20-30	0.4
Gaspard *et al*. (1990)	*M. incognita*	172 cm^3^	800 J2 to 1500 J2	3 mon	500 DD20-30	0.4
Ploeg and Maris (1999)	*M. incognita*	1 L	2,000 J2	Varied	16.2, 19.5, 25.0, 30.0, and 35.4°C.	0.02–1.6
Singh and Khurma (2008)	*M. incognita*	2 L	0, 200, 400, and 600 J2	8 wk	N/A	(avg # galls 13,19,26) (avg # egg masses 6, 11, and 17)
Silva *et al*. (2019)	*M. incognita*	2500 cm^3^	5,000 eggs	8 wk	25°C	17.2
Cristóbal-Alejo *et al*. (2021)	*M. incognita*	5 kg	2,500 eggs and 1,625 J2	92 d	30 ± 2°C	1700 eggs/g of root, 45 females per gram of root, 150 galls per plant
Yeon *et al*. (2021)	*M. incognita*	9.5 cm d	10,000 eggs	6 wk	25 ± 5°C	3.9 RF/9 gall index
Vega-Callo *et al*. (2021)	*M. incognita* and *M. hapla*	3 kg	5,000 eggs and J2	6 mon	25 ± 5°C	*M. incognita*: (8.4) *M. hapla* (2.53)
Kankam and Adomako (2014)	*Meloidogyne* spp.	1 L	500, 1,000, and 2,000 J2	12 wk	23.4–34.5°C	1.2–1.7
Wram and Zasada (2020)	*M. incognita, M. chitwoodi*, and *M. hapla*		1,500 J2	15 wk	26/18°C d/night	*M. incognita*: (61.2), *M. chitwoodi*: (25.5) *M. hapla* (32.2)
El-Sherif *et al*. (2007)	*M. incognita*	850 g	250,500, and 1,000 eggs	45 d	23 ± 4°C	1.1–1.5

a Either amount of soil or volume of the pot was reported. When only dimensions of pot were provided, abbreviations used are: h = height, d = diameter.

b Life stage of inoculation type was either eggs or J2

c Reproduction factor (RF = final population density/initial population density) was provided in the study or calculated from information in the study when possible. If not possible, other measures of reproduction are reported.

### Experiment 2: Impact of life stage and duration of experiment on M. incognita reproduction

The experiment consisted of 32 experimental units (pots), and was a factorial design with the following factors: 2 life stages (egg and J2) and 4 assay durations (5 wk, 6 wk, 7 wk, and 8 wk post inoculation) (Table 2). All factor combinations were replicated four times, and pots were arranged in a completely randomized design; the experiment was conducted twice. The experiment was conducted in 10 cm^2^ 0.6 L pots containing 500 g of steam pasteurized 1:1 sand:loam soil. The plants were inoculated with 1.0 *M. incognita* egg/g soil. Planting of tomato, nematode inoculation, and maintenance of the experiment was conducted as described for Exp. 1. At each takedown date, the plants were destructively harvested, and the eggs extracted from roots as described above.

### Experiment 3: Impact of duration of experiment on M. hapla and M. chitwoodi reproduction

Two identical but separate experiments were conducted for *M. chitwoodi* and *M. hapla*. Each experiment consisted of 16 experimental units (pots). A single factor was considered, that is, assay duration (5 wk, 6 wk, 7 wk, and 8 wk post inoculation) (Table 2). All treatments were replicated four times and pots were arranged in a completely randomized design; the experiment was conducted twice. The experiment was conducted in 10 cm^2^ 0.6 L pots containing 500 g of steam pasteurized 1:1 sand:loam soil. Planting of tomato, nematode inoculation, and maintenance of the experiments was conducted as described for Exp. 1. Plants were inoculated with 1.0 egg/g soil of each *Meloidogyne* spp. At each takedown date, the plants were destructively harvested, and the eggs extracted from roots as described above.

### Statistical analysis

Data was assessed separately for each experiment. For Exp. 1, the evaluation and prediction of a three-way interaction between life stage, inoculation density, and volume of soil was determined of the highest interest. Initially, a visual investigation of the data using a series of bar graphs was performed, followed by a linear model of the data. Using a backward-step determination method, the analysis started with the three-way interaction present, but promptly removed this interaction due to lack of significance. Three more models were attempted, one with all three two-way interactions, the next without an interaction between pot size and inoculation density, and the final only containing an interaction between life stage and inoculation density, and the three variables of interest. To determine the degree of significance between each treatment, Tukey's ad-hoc tests were performed on the model results.

For Exp. 2, bar graphs were initially produced to visually inspect the data. It was determined that assay duration should be considered a categorical variable as the plants were taken down at discrete intervals post-inoculation rather than along a continuous time scale. With this adjustment, the data was assessed for the best model to explain the data, and determine the optimized assay duration based on the life stage used for inoculation. To determine the degree of significance between each treatment, Tukey's ad-hoc tests were performed on the model results.

Exp. 3 followed a similar format of assessment as Exp. 2. RF values across species were not assessed, but each was assessed for a species-specific optimized assay duration. The best model and optimized assay duration were assessed for each *Meloidogyne* spp. using a mixture of linear model assessments and bar graphs. To determine the degree of significance between each treatment, Tukey's ad-hoc tests were performed on the model results. Statistical analyzes were done using R 4.0.4 (R Core Team, 2021) using the “ggplot2” (v3.3.3; Wickham, 2016), the “car” (v3.0-10; Fox and Weisberg, 2019), the “effects” (v4.2-0; Fox and Weisberg, 2019), the “grid” (v3.6.1; R Core Team 2021), and the “readxl” (v 1.3.1; Wickham and Bryan, 2019) packages.

## Results

### Experiment 1

**Figure 1:**
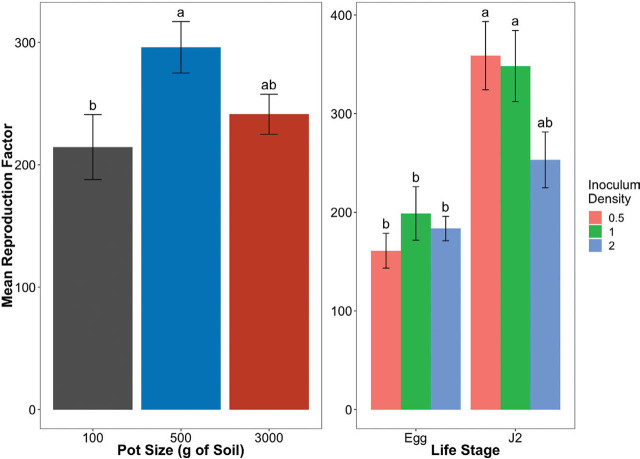
*Meloidogyne incognita* reproduction factor (RF = initial density/final density) on tomato as influenced by life stage, inoculation density, and/or pot size. Letters of significance were determined using the Tukey ad-hoc test of treatments. (A) Reproduction factor values by pot size. Darkening shade of the bars delineates the increasing pot size from 100 g to 3,000 g. (B) Reproduction factor values by life stage and inoculation density, regardless of pot size.

Following a backwards model assessment, starting with the most complicated model and removing the least significant variable at each step, a final model was reached to investigate the data from Exp. 1. The final model included all three variables of life stage, inoculation density, and pot size, but only had one interaction between life stage and inoculation density. There was strong evidence that there was a difference in *M. incognita* RF values between pot sizes (Fig. 1; *P* = 0.0092). On average, pots containing 500 g soil had a RF value 81.5 U greater than pots containing 100 g soil (*P* = 0.0027), while pots containing 3,000 g soil had a RF value of only 27.0 U greater than pots containing 100 g soil (*P* = 0.3167). A significant difference was observed between the pots containing 500 g and 100 g soil, but the same was not observed between pots containing 100 g and 3,000 g soil, or between 500 g and 3000 g soil. There was also strong evidence that there was a difference in *M. incognita* RF values between the life stages (Fig. 1; *P* = 2.525e^−09^). Pots inoculated with *M. incognita* J2 resulted in a RF value 197.8 U higher than pots inoculated with eggs, regardless of pot size or inoculation density (*P* = 5.81e^−07^). There was no interaction between life stage and inoculation density (*P* = 0.0557).

### Experiment 2

**Figure 2:**
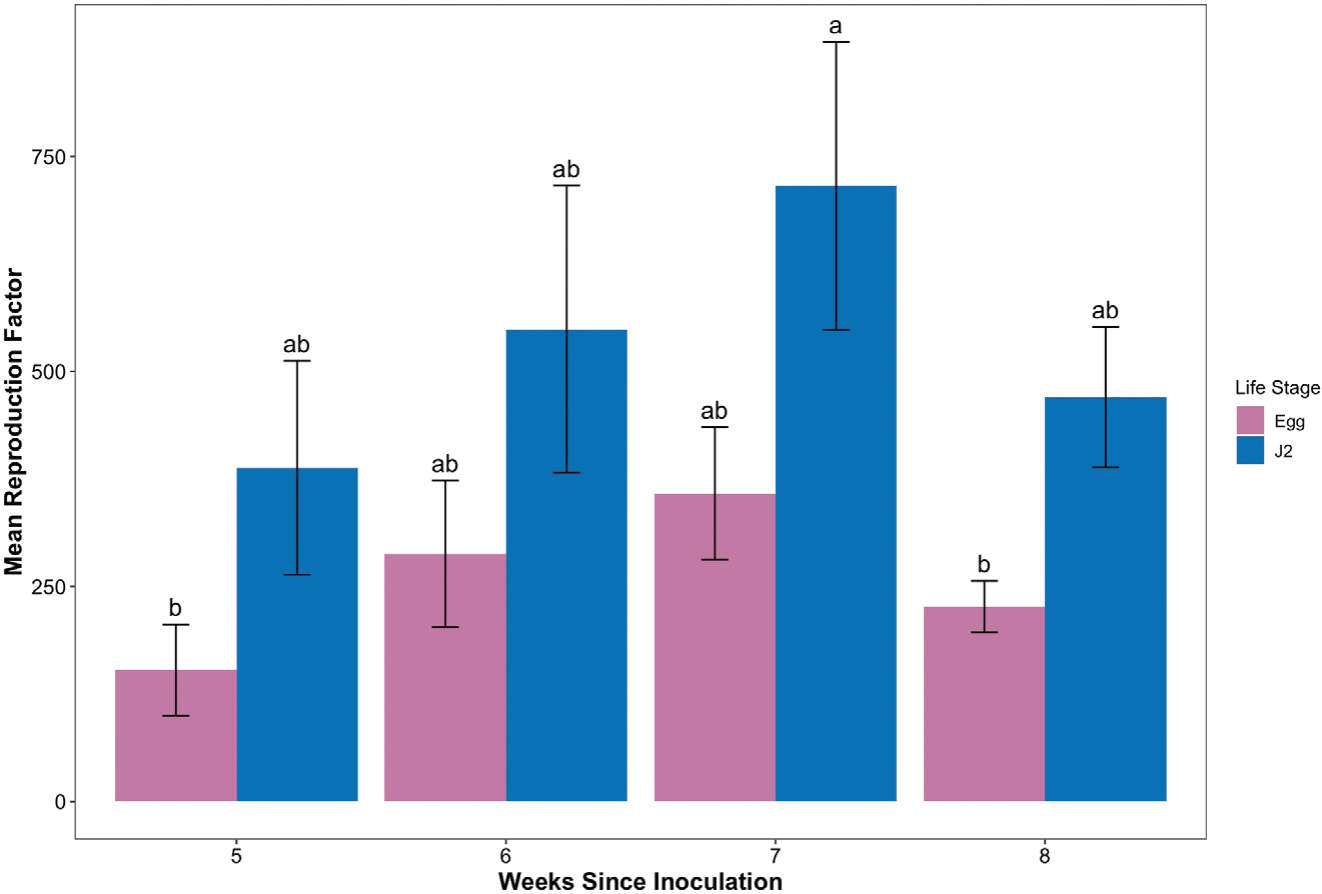
*Meloidogyne incognita* reproduction factor (initial density/final density) on tomato over time when inoculated with J2 or eggs. Letters of significance were determined using the Tukey ad-hoc test of treatments.

**Figure 3:**
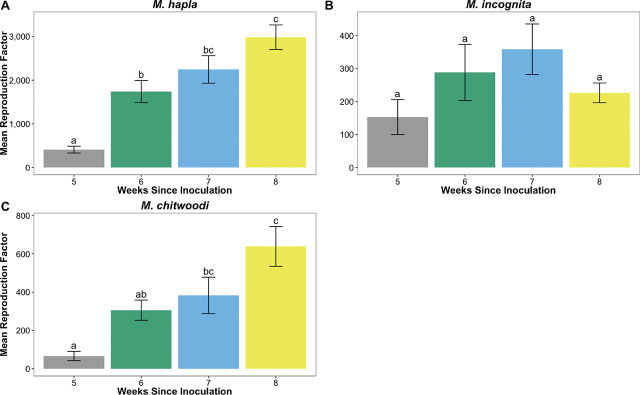
Reproduction factor (initial density/final density) values of three *Meloidogyne* species over time on tomato. Letters of significance were determined using the Tukey ad-hoc test of treatments. (A) *M. hapla*, (B) *M. incognita*, and (C) *M. chitwoodi*.

In the final model there was no interaction between assay duration and life stage (Fig. 2; *P* = 0.6745). There was strong evidence that there was a difference in RF values between *M. incognita* life stages (*P* = 0.0005). Similar to Exp. 1, inoculation with *M. incognita* J2 resulted in a RF value 274.3 U greater than when eggs were used as inoculum, regardless of assay duration (*P* = 0.0005). There was no difference in *M. incognita* RF values due to assay duration, regardless of life stage (*P* = 0.0907). There was a trend for higher RF values up to 7 wk after inoculation (Fig. 3).

### Experiment 3

Assay duration significantly impacted RF values of *M. hapla* (*P* = 6.466e^−07^). At 6 wk, 7 wk, and 8 wk post inoculation, RF values were 1,330 U, 1,836 U, and 2,576 U greater than RF values at 5 wk post inoculation, respectively (Fig. 3; *P* < 0.0001). *M. hapla* RF values were significantly greater at 8 wk post inoculation compared to 6 wk post inoculation (*P* = 0.0007). There was also a significant impact of assay duration on RF values of *M. chitwoodi* (Fig. 3; *P* = 0.0001). While RF values were similar for *M. chitwoodi* at 5 wk and 6 wk post inoculation (*P* = 0.3920), at 7 wk and 8 wk post inoculation RF values were 316.7 U and 572.4 U greater than at 5 wk post inoculation, respectively (*P* < 0.006). *M. chitwoodi* RF values were the highest at 8 wk post inoculation, however, this was not different from those observed at 7 wk post inoculation (*P* = 0.3294).

## Discussion

The objective of this study was to improve the methodology of greenhouse reproduction assays for *Meloidogyne* spp. to optimize RF values through efficiently utilizing inoculation resources, soil or supplies, and time. The impacts of pot size, life stage, inoculation density, and assay duration on *Meloidogyne* spp. reproduction are discussed below.

When attempting to optimize *M. incognita* reproduction, one of the first variables examined was pot size. In Exp. 1, contrary to intuition, a larger pot size did not increase RF values. This could be attributed to the fact that roots in smaller pot sizes are more densely compacted, so migrating *M. incognita* J2 are more likely to come across a root to invade in a small pot than a large pot. This pattern could disappear if the assay duration was increased. In a longer assay, plants in a large pot would have the space and nutrients available to produce more roots, and therefore, be able to sustain more nematodes. However, with a pot containing 500 g soil and a relatively short assay time (8 wk), we still observed robust RF values for *M. incogntia* of >250. For most research questions, it may not be necessary to increase the length of an assay to utilize a larger pot, unless maximizing root size is the goal of the experiment being conducted.

One of the critical considerations for starting a reproduction assay is choosing which life stage to use in an experiment. In Exps. 1 and 2, we assessed the effects of inoculum life stage on *M*. *incognita* reproduction. In Exp. 1, all but the highest inoculation density using J2 as an inoculation type resulted in 1.8- and 2.2-fold higher *M. incognita* RF values than eggs. This same pattern was observed in Exp. 2, although not statistically significant; inoculation with J2 resulted in RF values between 1.9- and 2.5-fold higher than eggs at each experiment takedown week. This increase in reproduction potential of *M. incognita* J2 reflects the ability of J2 to readily invade plant roots when applied to a root system. Whereas, eggs must molt into a J1 and J2, and break through the eggshell before being able to invade a root. In addition to the longer time to invasion, only a fraction of eggs when applied to a root system will be ready to proceed through these developmental stages. Vito *et al*. (1986) found that, at most, 40% of *M. incognita* eggs hatched after 3 wk incubation at 25°C after being extracted from egg masses. The small proportion of eggs that were able to hatch could contribute to the disparity in RF values observed between *M. incognita* J2 and eggs in the current study. While eggs are the easiest inoculation type to acquire for a reproduction assay, they will not always lead to the greatest RF, an important consideration for experimental design.

In addition to the life stage of the inoculum, inoculum density also plays a role in *M. incognita* reproductive capacity on a plant. Three inoculation densities of both eggs and J2 were examined for their effects on *M. incognita* reproduction on tomato. In Exp. 1, inoculation density did not have an impact on RF values. This is consistent with other inoculation density studies. Kankam and Adomako (2014) evaluated three different inoculation densities of *M. incognita* J2, 500, 1,000, and 2,000, with resulting RF values after 12 wk of 1.7, 1.3, and 1.2, respectively. El-Sherif *et al*. (2007) also observed RF values of 1.14, 1.17, and 1.48, which are relatively consistent values, when using starting inoculation densities of 250, 500, or 1,000 *M. incognita* eggs, respectively. However, when inoculation density varied by 10-fold, this relationship changed. Inoculation densities of 1,000 and 10,000 eggs of *M. hapla* and *M. chitwoodi* were used to evaluate reproduction at 25° and 30°C (Santo and O’Bannon, 1981). RF values at the 1,000 eggs inoculation density for *M. chitwoodi* at 25° and 30°C were on average 442.8 and 12, respectively, while with a starting inoculation density of 10,000 eggs *M. chitwoodi* RF values were 56.2 and 6.9 at 25° and 30°C, respectively. This pattern held true for *M. hapla*; RF values for the 1,000 egg density were 313.6 and 175.6 at 25° and 30°C, respectively, whereas at the 10,000 egg density RF values were 27.5 and 17.1 at 25° and 30°C, respectively, a greater than 10-fold difference. As inoculation densities increase, the capacity for the plant to support high levels of *Meloidogyne* spp. parasitism decreases due to competition among the individual nematodes for limited resources, damage and reduced capacity of nutrient uptake by the plant due to infestation, and environmental conditions (Perry *et al*., 2009). The relationship between inoculation density and RF is best represented by a sinusoidal curve (Oostenbrink, 1966). This sinusoidal relationship could explain why, in the present study, there were no significant differences in RF values due to inoculation densities. It is important to consider this relationship when designing a greenhouse reproduction assay; using a starting inoculation level that is beyond the carrying capacity of the root system of interest could obscure the results of the experiment.

Assay duration was the final component of the reproduction assay methodology that was tested in this study, and this was considered for three *Meloidogyne* spp. Optimum RF values for *M. incognita*, *M. hapla*, and *M. chitwoodi* were obtained by 7 wk post inoculation. For *M. chitwoodi* and *M. hapla*, RF values were the highest at 8 wk post inoculation, but we found no statistical differences in RF values at 7 wk and 8 wk post inoculation. For *M. incognita*, RF values decreased at 8 wk compared to the mean RF value at 7 wk post inoculation. These two trends reflect the life cycle lengths of these three *Meloidogyne* spp. at the greenhouse temperature used in this experiment of 26°C d/18°C nights. For *M. incognita*, the decrease in RF value at 8 wk was most likely due to the start of a new reproduction cycle. J2 hatched from eggs to invade new tomato roots resulting in fewer eggs in the system. At 25°C, *M. incognita* can complete a life cycle in just 28 d (Ploeg and Maris, 1999), or at 21°C, completion occurs in 37 d (Perry *et al*., 2009). Eggs were used as the inoculation type for this experiment, and accounting for the variability in hatching time and invasion time that can occur (Perry *et al*., 2009), 7 wk corresponded well to the peak of reproduction for *M. incognita*. *M. chitwoodi* has an optimum developmental temperature of 20°C (Santo and O’Bannon, 1981), 6°C lower than the average temperature used in this study. This could explain why this nematode took longer to develop, and the assay durations examined in this study might not have captured the peak reproduction of this species. *M. hapla* also prefers cooler temperatures, between 20°C and 25°C (Griffin *et al*., 1986), which could also explain why RF values continued to increase after 7 wk. Using the same populations of these three *Meloidogyne* species, a similar greenhouse assay was conducted by Wram and Zasada (2020), using a starting inoculum of 1,500 J2 and an assay duration of 15 wk at 26°C d/18°C nights. In that study, *M. incognita* had a RF >2 times of that of *M. hapla* and *M. chitwoodi*, corroborating that *M. hapla* and *M. chitwoodi* develop slower at the greenhouse temperatures used in this study, as well as the current study.

In conclusion, a moderate pot size (500 g of soil) inoculated with *M. incognita* J2 resulted in maximum RF values. The duration of the assay will depend on the *Meloidogyne* spp. in question, with longer duration assays required for *M. hapla* and *M. chitwoodi* than for *M. incognita.*
